# Security Control of Denial-of-Service Attacks in Cyber-Physical Systems Based on Dynamic Feedback

**DOI:** 10.1155/2022/5472137

**Published:** 2022-06-13

**Authors:** Yihe Wang, Bo Hu, Xiao Pan, Tingting Xu, Qiuye Sun

**Affiliations:** ^1^Northeastern University, College of Information Science and Engineering, Shenyang 110000, China; ^2^State Grid Liaoning Electric Power Co., Ltd., Shenyang 110000, China; ^3^Economic and Technology Research Institute State Grid Liaoning Electric Power Co., Ltd., Shenyang 110015, China; ^4^State Grid Liaoning Electric Power Co., Ltd., Metering Center, Shenyang 110000, China

## Abstract

With the integration and development of multiple disciplines, as well as a large amount of research on multilevel interconnection networks, wireless sensor networks have become a new access point and have attracted widespread attention worldwide. However, limited by the characteristics of the wireless sensor network itself, the energy problem has repeatedly restricted its function, which needs to be solved urgently. This paper mainly improves the Geographical and Energy-Aware Routing (GEAR) based on cyber-physical systems and energy-aware routing protocols, which reduces the resource consumption of network nodes, achieves network power balance, and further extends the system life cycle. Although the open sharing of the network layer makes CPS more efficient in actual use, it also increases the risks faced by CPS. Among these potential risks, cyberattacks have become the main security problem that CPS must solve with the greatest destructive power. Among them, Denial of Service (DoS) is the simplest and most destructive type of network attack. We can allow the system to reject normal requests for sending and receiving data using the network resources of the communication channel, thereby performing security control. From the perspective of control, this paper studies the security control of CPS in DOS attacks. Firstly, considering the diversity of CPS packet loss factors, assuming that there is an upper limit on the probability characteristics of the total number of packet loss development, the Markov process of inherent packet loss and the Markov process of DoS attack satisfying this constraint are analyzed, and the above two independent packet loss factors are described on this basis. Two independent packet loss factor descriptions are given in the following. In this paper, through the research of wireless sensor networks, it is applied to the cyber-physical system and the cyber-physical system is further improved.

## 1. Introduction

With the rapid development of ICT technology and automation control technology, the distribution network has been coupled to the information system and the physical distribution network (CPS distribution network) of the physical system. While the information system supports the stable operation of the physical system, it also brings security risks. For example, there is an attack on the information system, such as an attack on the network, which leads to the failure of services or performance functions and damages the safe and stable operation of the CPS distribution network [1]. Therefore, it is necessary to use effective and reasonable methods in a large-scale CPS distribution network environment to evaluate safety control. Among them, the wireless sensor network is a network composed of a large number of sensor nodes with functions such as calculation, analysis, and fusion [2]. Compared with other networks, the application of wireless sensor networks in the communication and network fields is very different; that is, wireless sensor networks can maintain normal network operation without a fixed base station [3]. Real-time data collection is performed through the sensor nodes on the network, data preprocessing operations are performed after the data collection is completed, then the information is sent to other nodes using the cyber-physical system, and the nodes can use Bluetooth and ZigBee technology to complete communication and perform data transmission and finally receive data from the user [4, 5]. Next, consider the various attack channel options in a Denial-of-Service (DoS) attack. On the one hand, the attacker can attack the output channel or the return channel, thereby modeling the attacked CPS as a switching system with three situations; on the other hand, the Denial-of-Service (DoS) attack is continuously random, and the attacked CPS can be modeled as a nested switching system based on these three types of subsystems. The number of subsystems in this switching system is dynamically variable. For insecure switching systems that are affected by DoS attacks and change in real time, the controller continues to calculate multiple control variables during the DoS attack and sends them together when it can transmit data to compensate for the information. Then, the CPS exponential stability condition is given, and an output dynamic feedback controller is designed to meet the condition [6]. The inverted pendulum model in the network verifies the effectiveness of the proposed method. Finally, combined with the actual situation, the impact of smart DoS attacks on CPS was investigated, and a cross-sectional design of the security control method was carried out. The so-called DoS intelligent attack means that the attacker weighs the cost of the attack under energy constraints and chooses the strategy that is most beneficial to him [7]. For this reason, the optimal strategy of the offensive and defensive part of the game is analyzed, and the optimal packet transmission rate of the strategy for the lower CPS network layer is obtained [8]. In order to analyze the exponential stability of the physical layer, a sliding mode control method is proposed according to the obtained CPS exponential stability conditions [9]. The example simulation verifies that the designed game strategy and control method can ensure the stability and safety of CPS [10].

## 2. Related Work

The literature introduces the incidence matrix CPS modeling technology of the distribution network. This paper uses an incidence matrix-based modeling technology to accurately model the layer structure of the distribution network CPS and allocate the physical entity layer, the physical information coupling layer, and the information system layer to form the CPS unit model of the entire distribution network [11]. The literature introduces that a cyber-physical system refers to the system formed by the close connection of computer, communication, and physical process [12]. The system can integrate perception, communication, and computing technologies into physical devices, thereby fully realizing distributed identification, information transmission, intelligent information processing, feedback, and real-time control [13]. Therefore, cyber-physical systems are used in many fields, including chemicals, power grids, water distribution, automobiles, and smart homes. The transmission of information is the most basic function in a cyber-physical system. Wired or wireless transmission equipment is subject to various attacks from the information space, resulting in errors or failures in information transmission, resulting in control system performance or instability [14]. Therefore, the security issues of cyber-physical systems have attracted more and more attention from researchers, and the results of related investigations have also expanded from the initial communication field to the control field [15]. The security control theory is based on the attack model, so it is very important to establish a suitable attack model. In previous studies, the most common attacks are divided into two categories: the first is a Denial-of-Service (DoS) attack, and the other is a spoofing attack. False data injection (FDI) attack is a typical example of a spoofing attack. In recent years, the research on cyber-physical system security control in these two types of network attacks has received more and more attention and has made certain progress, but there are still many shortcomings [16]. For example, the existing research methods for the elastic control of cyber-physical systems in Denial-of-Service attacks do not consider the unknown state transition probability of the information layer, and the research on attack behavior is relatively simple; that is, most research only considers the situation of a single type of attack: cyber physics system control issues. The literature introduces the security problems of CPS under DoS attacks and proposes a game-based H control strategy algorithm [17]. On the one hand, consider the zero-sum game of control performance and use performance counters to design mixed information layer strategies; on the other hand, consider the zero-sum random game of offensive and defensive strategies, and use the optimal hybrid network strategy to design the physical system controller. These two procedures merge together to form a network-based security control investigation method. The literature introduces a design algorithm with a limited time range. The hybrid attacks mentioned by H include DoS attacks on the communication channel between sensors and controllers and FDI attacks on sensors and actuators. Considering that hybrid attacks will cause more serious damage to the physical performance of CPS, the relationship between the attack injection signal and the controlled output is investigated, and the controller gain is designed to make the closed-loop system meet the performance bar H in the finite time domain. The purpose of this paper is to solve two backward coupled recursive Riccati difference equations under certain conditions, and to reduce the impact of attack input signal on linear quadratic Gaussian performance in the worst case [18, 19].

## 3. Security Control of the Wireless Sensor Network and Denial-of-Service Attacks

### 3.1. Wireless Sensor Network

**Figure 1:**
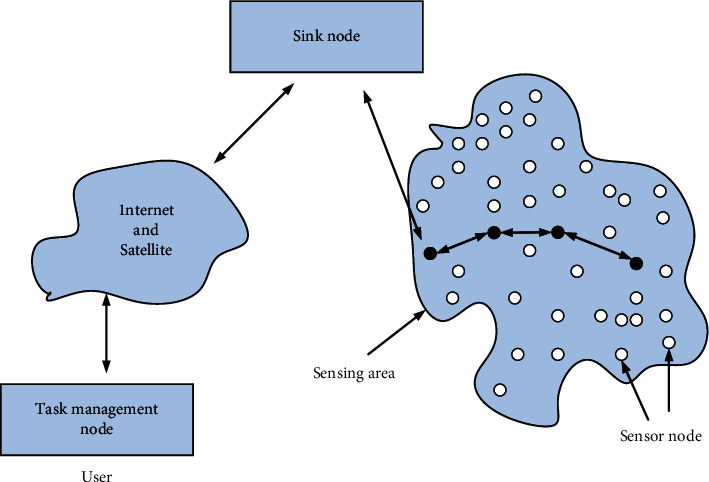
Wireless sensor network architecture.

The wireless sensor network (WSN) is composed of sensor nodes and sink nodes distributed in the detection area. However, a large number of ordinary sensors are randomly scattered in a certain area to collect monitoring data. Monitoring data is generally sent from the source node, and the intermediate node receives the data as a relay, then sends it to the next node, and finally sends it to the last node. The receiving node uploads the monitoring data collected by each node in the sensor network to the base station, which is connected to the internal and external networks of the sensor system to provide users with the required services. The system architecture is shown in Figure 1.

The network is mainly composed of data collection network, information transmission network, and task management network. Its usual working method is distributing ordinary sensor nodes randomly in the monitoring area and adopting self-organization theory to organize an adaptive network to realize the data monitoring function of the corresponding area. Internally, each node has the function of collecting data and selecting the transmission path. The collected data is usually sent to the receiving node in a multihop manner between nodes. When forwarding data to the receiving node, the receiving node can undergo multiple merging processes to improve the accuracy and credibility of the collected information. WSN can be connected to an external satellite network or the Internet through a base station.

It can be seen from the above process that the success of decoding is closely related to the encoding vector. Table 1 shows the relationship between finite field assignment and unrelated linear probability. If the finite field is *qF* = 28, the node failure probability is 0.004, which meets the expected requirements. Therefore, the median finite field of this paper is 28, as shown in Table 1.

### 3.2. Cyber-Physical System

The CPS architecture generally consists of two main layers: the network layer and the physical layer. The current state of CPS includes sensor output variables and controller control variables. In CPS, the controller calculates the offset between the output and the corresponding set point. After the controller calculates the offset, it calculates a new control value and sends it to the corresponding actuator to make the bit transmission layer closer to the preset value.

Broadly speaking, the CPS process can be broken down into the following stages: monitoring, networking, computing, and driving. The network layer usually uses industrial protocols such as DNP3, 61850, and Modbus to communicate with devices on the physical layer. Different types of CPS functions depend on the combination and application of several key layers. For different application fields, it is generally necessary to consider which key functions are used and to what extent. Based on consideration of different levels, CPS can be divided into seven basic levels: from the physical layer to the application layer.

Physical layer: the physical layer forms the foundation of the CPS architecture. The physical layer consists of sensors and actuators connected to each other through a wireless or wired network, for example, 2G/3G/4G, WIFI, ZigBee, Bluetooth, RFID, and wired technology. This layer is mainly used to connect ZigBee, Bluetooth, and other systems to the network (as a router). Devices at this level usually do not have a lot of memory or processing power, and most attacks at this level are external.

Link layer: the link layer can create, send, and receive data frames. This layer serves the needs of the network layer and uses the services of the physical layer to receive and send data packets. The data link layer is divided into a sublayer for logical channel management (LLC) and a sublayer for media access control (MAC). LLC provides network layer services, and the MAC sublayer manages access to the shared physical environment. Attacks on this layer will cause the MAC address to be disturbed, which will result in invalid device identification.

Network layer: at this layer, the frames provided by the data link layer are formed into data packets, the header of the network layer is encapsulated in the packet, and the packet contains logical address information. Malicious attacks can invalidate sensors and actuators, leading to changes in information sources and physical layer failures.

Transport layer: at this layer, data packets are broken down into small parts. The most common transport layer protocols include TCP, UDP, and ICMP. Attacks on this layer slow down the network equipment and cause service interruption.

Session layer: the session layer manages sessions. It monitors the sequence of messages transmitted over the network and inserts tags into long messages when errors occur.

Presentation layer: the presentation layer coordinates the representation of data in the interaction of two application processes. An example is the Secure Sockets Layer (SSL) protocol, which enables the exchange of secret messages for the application layer protocol of the TCP/IP stack.

Application layer: the application layer includes different areas. This layer stores, analyzes, and updates information obtained from previous layers. We can view the control decisions we make through the virtual interface. Data protection is the most important issue at this level.

### 3.3. Denial-of-Service Attack

Due to the vulnerability of CPS, attacks can invade the system in a hidden and unpredictable way through the network part. For example, an attacker can inject malware (such as viruses and worms) to interrupt computer programs or disable network components at the media access control level. In addition, the attacker can illegally access the monitoring center after receiving the encryption key to disrupt its normal operation. In other words, if CPS does not have adequate security protection for hardware or software policies, an attacker can arbitrarily destroy the state of the system in some way. In a model-based analysis framework, it is important to describe cyberattacks from the perspective of mathematical models.

DoS attack is an attack method that makes system resources unavailable. From a technical point of view, an attacker can fill a buffer in the user domain or the central domain, block the shared network medium to prevent the device from sending and receiving data, or change the routing protocol. At present, some mathematical models are used to quantitatively analyze system performance degradation caused by DoS attacks, such as queue model, Bernoulli model, and Markov model. Among them, the system attacked by the tail model can be transformed into a time-delay system, and traditional analysis methods can effectively solve the problem based on stability. A hierarchical, time-based recursive predictive control scheme is proposed to compensate for weak DoS attacks and packet loss under communication constraints. The optimal programming problem of DoS attack is to maximize the estimation error in the remote estimator with an intrusion detection system. Even though the physical mechanisms of DoS attacks and packet loss are different, their mathematical models in the Bernoulli framework are the same. Therefore, it makes sense to use typical packet loss analysis methods to check the performance of CPS systems affected by DoS attacks.

This document suggests a limited DoS attack model. As long as the duration of the DoS attack has a certain upper limit within a certain period of time, the DoS attack can be carried out in both directions. Under the continuous timing of this model, the timing of the attack can be described as follows. There are scalar *κD* ≥ 0 and *ρD* ∈ [0,1) such that

(1) $$\begin{array}{c} \left|A\left(0,t\right)\right|\leq \kappa_{D}+\rho_{D}t,\;t\geq 0. \end{array}$$

There are scalars *κF* ≥ 0 and *ρF* ∈ [0,1) such that

(2) $$\begin{array}{c} ℐ\left(0,t\right)\leq \kappa_{F}+\rho_{F}t,\;t\geq 0. \end{array}$$

A commonly used method is to define the game process at the physical layer and the network layer. Considering the CPS physical layer, the evolution of the system generally adopts the following model:

(3) $$\begin{array}{c} \dot{x}\left(t\right)=g\left(t,x,u,w,\eta \left(t,\alpha ,\beta \right)\right). \end{array}$$

Considering the remote state estimation problem of the network layer of DoS attacks, the CPS is modeled as a time-discrete linear system:

(4) $$\begin{array}{c} x\left(t+1\right)=Ax\left(t\right)+\omega \left(t\right),\\ y\left(t\right)=Cx\left(t\right)+\nu \left(t\right). \end{array}$$

**Figure 2:**
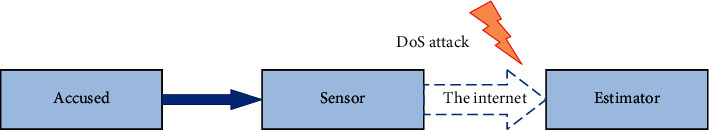
Estimation of the networked state of a DoS attack.

The network state estimation framework in this paper is shown in Figure 2.

## 4. Research on Cyber-Physical System Security Control Based on Wireless Sensor Networks and Denial-of-Service Attacks

### 4.1. Status Feedback Control of Cyber-Physical Systems under Denial-of-Service Attacks

CPS is a large and complex system with highly integrated and coordinated physical equipment and network resources. On the basis of the traditional network control system and the Internet of Things, the joint design of communication technology and control technology is emphasized, making CPS an important area of production and life, with a higher technical level and higher use efficiency. Similar to the network control system, although the introduction of the CPS network layer will cause accidental packet loss, it must also guard against various network attacks. These random or malicious unstable factors often cause system damage, become a reality in reality, and even lead to loss of life and property.

On the one hand, due to different network protocols and other interference factors that affect the transmission of data packets, accidental packet loss in the network system is inevitable. A large number of documents have proposed related control strategies and compensation methods for this type of packet loss. On the other hand, the CPS network layer is exposed to multiple attack methods. DoS attacks are currently the most widely investigated attack. This kind of attack is simple and easy to implement. When successful, it is actively consuming system resources, causing abnormal network services and causing a large amount of system packet loss. In fact, these two types of unstable factors usually coexist in CPS and are more destructive than when only one factor is considered. Therefore, one of the key issues that need to be resolved is how to fully consider the impact of the two on CPS, construct related models, and propose safety control methods.

This section introduces the CPS model with random packet loss and DoS attacks. In order to describe the characteristics of packet loss, the upper limit of the packet loss rate is assumed. First, according to the constraints, the Markov process of accidental packet loss caused by DoS attacks is given, and a unified attribute description is given based on the probabilistic independence of the two; secondly, in order to save communication resources, an event trigger scheme is introduced. The stability of event-driven CPS is further investigated, which provides conditions for the asymptotic stability of the system under random packet loss and DoS attacks; again, the state feedback controller is designed under asymptotic stability conditions; finally, a simulation example verifies the proposed effectiveness of the control method.

First, introduce the event trigger mechanism of the communication between the controller and the actuator. This mechanism is used to determine the data packet transmission time *τi* ∈ *N*0, *i* ∈ *N*0. For this, use the Lyapunov function *V*(*x*) = *x* TP*x*, where *P* > 0. Let *τ*0 = 0; we have

(5) $$\begin{array}{c} \tau_{i+1}≜\min\left\{t\in \left\{\tau_{i}+1,\tau_{i}+2,\ldots \right\}:t\geq \tau_{i}+\theta \text{ or }V\left(Ax\left(t\right)+Bu\left(\tau_{i}\right)\right)>\beta V\left(x\left(\tau_{i}\right)\right)\right\}. \end{array}$$

If there is a matrix *M* ∈ *Rm* × *n* and a positive definite matrix *Q* ∈ *Rn* × *n*, the parameters *β* ∈ (0,1), *φ* ∈ [1, *∞*)*β*, so that

(6) $$\begin{array}{c} \left[\begin{array}{cc} \beta Q & {\left(AQ+BM\right)}^{T}\\ AQ+BM & Q \end{array}\right]\geq 0, \end{array}$$

(7) $$\begin{array}{c} \left[\begin{array}{cc} \varphi Q & {\left(AQ\right)}^{T}\\ AQ & Q \end{array}\right]\geq 0. \end{array}$$

Using Schur's complement lemma, (6) and (7) can be written as

(8) $$\begin{array}{c} \beta Q-{\left(AQ+BM\right)}^{T}Q^{-1}\left(AQ+BM\right)\geq 0, \end{array}$$

(9) $$\begin{array}{c} \varphi Q-{\left(AQ\right)}^{T}Q^{-1}AQ\geq 0. \end{array}$$

Consider a networked inverted pendulum discrete system, and its actual model parameters are shown in Table 2.

**Figure 3:**
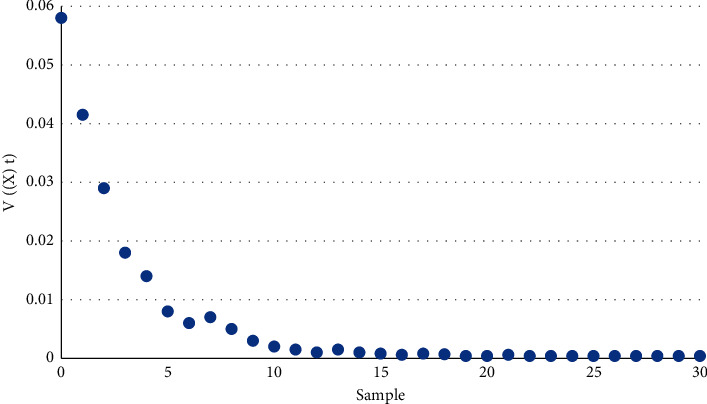
Sample trajectory of Lyapunov function *V*(•).

Figure 3 shows the same effect by depicting the sample path of the Lyapunov function.

### 4.2. Dynamic Output Feedback Control of Cyber-Physical Systems under Denial-of-Service Attacks

Although there are documents describing security control issues in DoS attacks, there are still many issues that have not been effectively resolved. Describing the impact of attacks on system performance in more detail is a challenging problem. The attack intensity and attack threshold are selected to describe the relationship between the attacker and the system performance, and the necessary and sufficient conditions of the Nash equilibrium are analyzed under the two-stage game model. However, under the interference of intelligent attackers, the system must passively select the best detection threshold corresponding to the attack intensity and cannot actively compensate for packet loss caused by DoS attacks. Based on the flexible control method of the switching system, a hybrid theoretical framework in which the controller switches between the network attacker and the defender based on the result of the competition is proposed. However, this classification method generally does not consider the impact of DoS attacks on the communication channels of the system.

Based on the above discussion, the purpose of this section is to develop a dynamic output feedback controller to solve the security problem of CPS under continuous DoS attacks. Comparing the general classification of switching subsystems based on continuous DoS attack characteristics, this paper not only focuses on the impact of DoS attacks on different CPS communication channels but also focuses on the persistence of the attack. In fact, in view of this situation, the controller can be designed more effectively so that, in the event of a DoS attack, many planned future control inputs can compensate for the CPS condition. The most important contributions of this section can be summarized as follows:

1. Considering the temporal and spatial characteristics of DoS attacks, the CPS under DoS attacks is modeled as a two-level nested switching system, and the dynamic evolution process occurs between two adjacent transmission switching points.
2. Using the proposed nested exchange system model, the exponential stability of CPS under DoS attacks is analyzed. The designed dynamic output feedback controller can provide many future control inputs when data transmission is successful.

For a given parameter *ε*_*i*,*n*_ > 1, *μ* > 1, if there is a matrix *P*_*i*,*n*_, *Y*_*i*,*n*_, *Q*_*i*,*n*_ > 0, *Q*_*i*,*n*_ = *P* − 1_*i*,*n*_ (*i* ∈ *M*, *n* ∈ *L*, *L* is a finite set and depends on the maximum number of consecutive DoS attacks. If *i* = 1, then *n* = 0). The following inequality applies:

(10) $$\begin{array}{c} \left[\begin{array}{cc} -P_{i,n} & \varepsilon_{i,n}K_{i,n}^{T}\\ {}* & -Q_{i,n} \end{array}\right]<0, \end{array}$$

(11) $$\begin{array}{c} P_{a,\alpha }<\mu P_{b,\beta }\left(\forall a,b\in M;\forall \alpha ,\beta \in L\right), \end{array}$$

(12) $$\begin{array}{c} \rho =\max\left\{\varepsilon_{i,n}^{-2}\mu |i\in M,n\in L\right\}<1. \end{array}$$

Equation (10) is a nonlinear matrix inequality. Multiplying diag{*I*, *P* − *1*_*i*, *n*_} to the left and right on both sides of inequality (10), respectively, and obtaining equation (9) is equivalent to the following matrix inequality:

(13) $$\begin{array}{c} \left[\begin{array}{cc} -P_{i,n} & \varepsilon_{i,n}K_{i,n}^{T}\\ {}* & -P_{i,n}^{-1} \end{array}\right]<0. \end{array}$$

Substituting the expressions in equations (6), (7), and (8) into (9), we can get

(14) $$\begin{array}{c} \left[\begin{array}{cc} -P_{i,n} & \varepsilon_{i,n}A_{1,0}^{T}A_{i,1}^{T}\ldots A_{i,n-1}^{T}A_{i,n}^{T}\\ {}* & -Q_{i,n} \end{array}\right]<0. \end{array}$$

**Figure 4:**
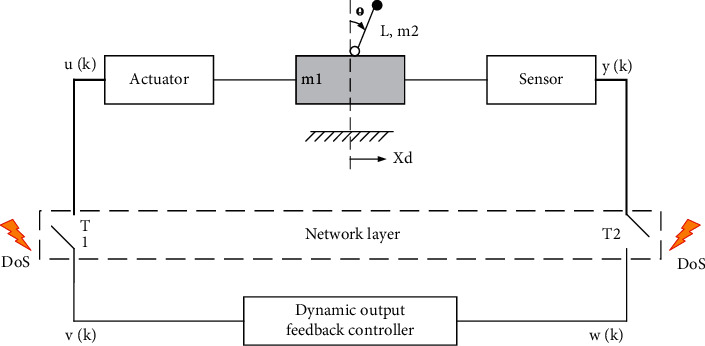
Networked inverted pendulum system.

Consider the inverted pendulum problem of a car as shown in Figure 4.

### 4.3. Sliding Mode Control of Cyber-Physical Systems under Denial-of-Service Attacks

In production and real life, the way in which a DoS attacker executes an attack is usually flexible and changeable. Although the previous section considered the randomness of DoS attacks in space and the continuity in time to a certain extent, most of the current attack vectors must measure the costs and benefits of attacks and defenses when security incidents occur. From this perspective, the existing literature generally uses game theory to solve such problems. For the CPS security game under DoS attacks, the existing research mainly combines the results of the game with the secondary performance cost index or the physical layer H performance index to analyze the flexibility of the system and rarely combines the game strategy with the security of the system. The switching systems control the cross-layer design method. In fact, the CPS attacked by DoS can be regarded as a switching system, which includes two subsystems: interference and noninterference. If the interference signal-to-noise ratio (SINR) is introduced as the performance measurement index of the CPS network layer, the average residence time is an important index for analyzing the stability of the handover system, which can be determined by the network. CPS is a measure of the level of competition results, which provides ideas for the CPS security cross-layer design in this section.

Different from the continuous random DoS attacks in the previous section, this section discusses CPS security control under the influence of smart DoS attackers. By combining the needs of the attacker and the benefits of the system, the game process is described as a Stackelberg game with a master-slave relationship. The result directly affects the size of the SINR and ultimately determines the packet transmission rate on the CPS channel under the equalization condition. Based on the analysis of the optimal strategy of attack and defense considering the maximum utility of the CPS network layer and the exponential stability of the physical layer, a discrete sliding mode control design method based on switching systems, Lyapunov, and complementary cone function linearization is proposed. Finally, the effectiveness of the control strategy is verified by experimental simulation.

In this paper, DoS attacks are considered malicious errors. In order to maintain the data packet transmission rate of the CPS wireless communication network, the concept of SINR is introduced:

(15) $$\begin{array}{c} \gamma_{T}=\frac{\eta \alpha \psi }{\beta \nu +\sigma^{2}}. \end{array}$$

According to digital communication theory, the relationship between symbol error rate (SER) and SINR is generally

(16) $$\begin{array}{c} \text{SER}=2Q\left(\sqrt{\gamma_{Tb}}\right). \end{array}$$

The data packet transmission rate of the wireless communication network under the DoS attack is

(17) $$\begin{array}{c} r_{0}={\left(1-\text{SER}\right)}^{b+1}={\left\{1-2Q\sqrt{\frac{\eta \alpha \psi B_{b}}{f_{b}\left(\beta \nu +\sigma^{2}\right)}}\right\}}^{b+1}. \end{array}$$

The controlled object is described as the following discrete-time state-space model:

(18) $$\begin{array}{c} \left\{\begin{array}{c} x\left(k+1\right)=Ax\left(k\right)+Bu\left(k\right),\\ y\left(k\right)=CxS\left(k\right). \end{array}\right) \end{array}$$

Design a state estimator with the following form:

(19) $$\begin{array}{c} \left\{\begin{array}{c} \hat{x}\left(k+1\right)=A\hat{x}\left(k\right)+Bu\left(k\right)+L\left[\bar{y}\left(k\right)-\hat{y}\left(k\right)\right],\\ \hat{y}\left(k\right)=C\hat{x}\left(k\right). \end{array}\right) \end{array}$$

The above formula can be written as

(20) $$\begin{array}{c} \hat{x}\left(k+1\right)=\left\{\begin{array}{c} A\hat{x}\left(k\right)+Bu\left(k\right)-L_{0}C\hat{x}\left(k\right),\\ A\hat{x}\left(k\right)+Bu\left(k\right)+L_{1}Ce\left(k\right). \end{array}\right) \end{array}$$

For the attacker, the attacked system is the defender. It is reasonable to assume that the offensive and defensive teams seek the best strategy in the same game. In the Stackelberg designed in this section, the defender is regarded as the leader, and the attacker is regarded as the follower. SINR defines the services of both parties as follows:

(21) $$\begin{array}{c} J_{d}\left(\psi ,\nu \right)=\frac{\eta \alpha \psi }{\beta \nu +\sigma^{2}}-\kappa_{d}\psi ,\\ J_{a}\left(\psi ,\nu \right)=-\frac{\eta \alpha \psi }{\beta \nu +\sigma^{2}}-\kappa_{a}\nu . \end{array}$$

If the actions taken by others (strategy choices) are known or predictable, then the strategy that gets the most profit from the known or predictable actions is called the best response. Specifically, the optimal response of the defensive side and the offensive side is

(22) $$\begin{array}{c} \pi_{d}^{*}\left(\nu \right)=\underset{\psi \in {ℜ}_{d}}{\text{max}}J_{d}\left(\psi ,\nu \right),\\ \pi_{a}^{*}\left(\psi \right)=\underset{\nu \in {ℜ}_{a}}{\text{max}}J_{a}\left(\psi ,\nu \right). \end{array}$$

In this section, the inverted pendulum model of the network is used to verify the effectiveness of the proposed cross-layer design method, and the system model parameters given in the previous section are used here. The initial state is *x* (0) = [0.4 0.2 0.3 0.1] *T*, and the inherent sampling period of the system is set to *T* = 0.1 s. Let *λ* = 1.2, *ε*_0_ = 1.4, and *ε*_1_ = 0.3, as shown in Table 3.

At the network layer, the channel parameters of the communication network are shown in Table 3. Therefore, the CPS packet transmission rate depends on the Stackelberg game between the system and the attacker. According to the theorem, the optimal strategy pair of the Stackelberg game can be calculated as (7.65, 2.51).

**Figure 5:**
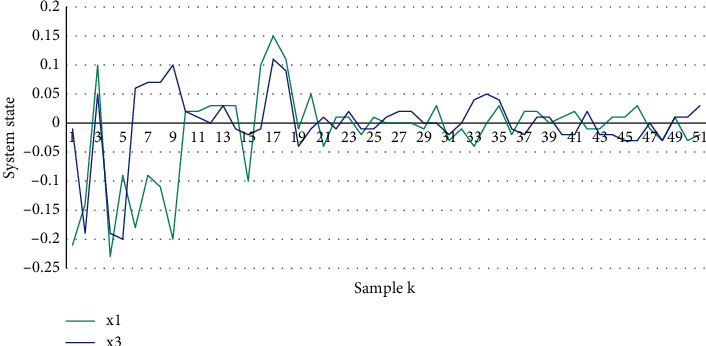
CPS status trajectory in a nongaming environment.

Under such an attack sequence, the trajectory of the CPS state is shown in Figure 5. It can be seen that, during the DoS attack, the graph fluctuates greatly. Obviously, the packet transmission rate of the CPS model that does not consider the Stackelberg game is low. In this case, the design of the physical layer controller alone cannot guarantee the safety of the system.

### 4.4. Analysis of Calculation Examples

The topological structure diagram of the physical side and the information side of the example model has been shown in this paper. The physical side contains 33 load nodes, two tie switches, and one distributed power supply.

Table 4 shows data such as the node load, importance, and number of users on the physical side of the network.

The parameters of the network importance and the number of users on the physical side are shown in Table 5.

**Figure 6:**
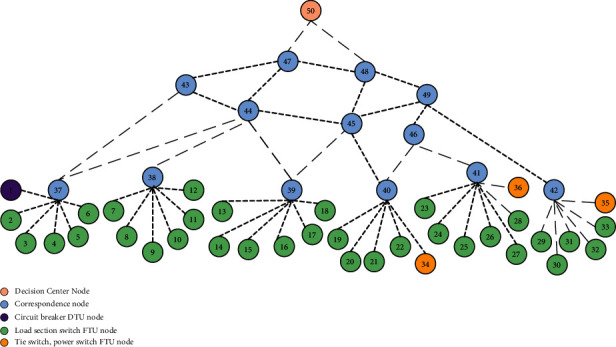
Information side network topology.

The information side network is shown in Figure 6, where node 1 is a circuit breaker node; nodes 2 to 33 are load section switch FTU nodes; nodes 34 to 36 are connection switches and circuit breaker nodes; nodes 37 to 49 are communication network nodes; nodes 50 is the decision center node.

## 5. Conclusion

With the progress of science and technology and the improvement of social production, the mutual penetration and integration of information space and physical space have directly promoted the development of CPS-related theories and applications. As a highly integrated and deeply coordinated control system, CPS has many similarities with networked control systems and the Internet of Things, but the CPS concept of “cooperative design and efficient operation” distinguishes it from the Internet of Things. Due to its unique technical advantages, CPS has been widely used in key areas of economy and people's livelihood, such as smart factories, smart transportation, telemedicine, power grids, and robotic systems. However, as CPS application technology matures, some problems have also been discovered. With the introduction of the network layer, CPS must not only solve the random instability factors of the physical layer but also face the risk of malicious attacks on the network layer, which seriously endangers the security of the system. This paper examines the impact of network attacks on the system from the perspective of control and communication and mainly examines the security control and cross-layer design of CPS under DoS attacks. For continuous random DoS attacks, passive control methods with state feedback or active compensation methods with dynamic output feedback control are proposed. In addition, for attackers with intelligent functions, game theory is used to design the network layer, and a sliding mode control strategy is proposed. This cross-layer design effectively guarantees the security and stability of CPS against DoS attacks.

## Figures and Tables

**Table 1:** The relationship between the size of the finite field and the linear irrelevant probability.

Linearly independent *p*	Finite field *F*	Linearly independent *p*	Finite field *F*	Linearly independent *p*	Finite field *F*
0.859406	2^3^	0.992126	2^7^	0.99951 1	2^1^
0.288788	2^1^	0.967773	2^5^	0.998043	2^9^
0.688538	2^2^	0.984131	2^6^	0.999022	2^10^
0.933595	2^4^	0.996078	2^8^	0.999756	2^12^

**Table 2:** Actual model parameters of the inverted pendulum system.

Parameter	Physical meaning	Value
M	Trolley quality	1.096 kg
*m*	Pendulum quality	0.109 kg
*b*	Friction coefficient of trolley	0.1 N/m/s
*I*	The length from the pivot axis of the pendulumrod to the center of mass of the rod	0.25 m
*J*	Pendulum inertia	0.0034 kg ^*∗*^ m^2^
9	Acceleration of gravity	9.8 N/kg

**Table 3:** CPS network layer parameters.

Parameter	Value
Channel gain *Q*	0.85
Interference gain 6	0.25
Background noise^*∗*^	0.1
Spread spectrum gain〃	3
Channel data rate *f*_*b*_	5
Channel width *B*_*b*_	1
Number of data packets b	7
Defender unit energy loss suit	2
Attacker unit energy loss heart	12

**Table 4:** Physical side network load data.

Node	Active power rate (kVA)	Reactive power rate (kVA)
1	100	60
2	90	40
3	120	80
4	60	30
5	60	20
6	200	100
7	200	100
8	60	20
9	60	20
10	45	30
11	60	35
12	60	35
13	120	80
14	60	10
15	60	20
16	60	20
17	90	40
18	90	40
19	90	40
20	90	40
21	90	40
22	90	50
23	420	200
24	420	200
25	60	25
26	60	25
27	60	20
28	120	70
29	200	600
30	150	70
31	210	100
32	60	40
33	60	20

**Table 5:** Parameters of network importance and number of users on the physical side.

Node	Importance	User number
1	10	0
2	8	200
3	8	80
4	4	50
5	7	60
12	2	50
13	5	10
14	3	200
15	3	100
16	3	80
23	10	40
24	9	20
25	2	20
26	2	60
27	2	40

## Data Availability

The data used to support the findings of this study are available from the corresponding author upon request.
